# Federated Learning-Based Predictive Traffic Management Using a Contained Privacy-Preserving Scheme for Autonomous Vehicles

**DOI:** 10.3390/s25041116

**Published:** 2025-02-12

**Authors:** Tariq Alqubaysi, Abdullah Faiz Al Asmari, Fayez Alanazi, Ahmed Almutairi, Ammar Armghan

**Affiliations:** 1 Department of Civil Engineering, College of Engineering, Northern Border University, Arar 73222, Saudi Arabia; 2 Civil Engineering Department, College of Engineering, King Khalid University, Abha 61421, Saudi Arabia; afsaeed@kku.edu.sa; 3 Civil Engineering Department, College of Engineering, Jouf University, Sakakah 72388, Saudi Arabia; fkalanazi@ju.edu.sa; 4 Department of Civil and Environmental Engineering, College of Engineering, Majmaah University, Majmaah 11952, Saudi Arabia; a.alaoni@mu.edu.sa; 5 Department of Electrical Engineering, College of Engineering, Jouf University, Sakakah 72388, Saudi Arabia

**Keywords:** deep learning, intelligent transportation, federated learning, privacy, security, vehicle communication

## Abstract

Intelligent Transport Systems (ITSs) are essential for secure and privacy-preserving communications in Autonomous Vehicles (AVs) and enhance facilities like connectivity and roadside assistance. Earlier research models used for traffic management compromised user privacy and exposed sensitive data to potential adversaries while handling real-time data from numerous vehicles. This research introduces a Federated Learning-based Predictive Traffic Management (FLPTM) system designed to optimize service access and privacy for Autonomous Vehicles (AVs) within an ITS. Moreover, a CPPS will provide strong security to mitigate adversarial threats through state modelling and authenticated access permissions for the integrity of vehicle communication networks from man-in-the-middle attacks. The suggested FLPTM system utilizes a Contained Privacy-Preserving Scheme (CPPS) that decentralizes data processing and allows vehicles to train local models without sharing raw data. The CPPS framework combines a classifier-based learning technique with state modelling and access permissions to protect user data against invasions and man-in-the-middle attacks. The proposed model leverages Federated Learning (FL) to enhance data security in collaborative machine learning processes by allowing updates that preserve privacy, enabling joint learning without exposing raw data. It addresses key challenges such as high communication costs, the impact of adversarial attacks, and access time inefficiencies. Using FL, the model reduces communication costs by 23.29%, mitigates adversarial effects by 16.1%, and improves access time by 18.95%, achieving significant cost savings and maintaining data privacy throughout the learning process.

## 1. Introduction

An Intelligent Transportation System (ITS) provides passengers with a secure, comfortable, intelligent travel experience. The process involves establishing a connection between drivers’ smartphones, roadside infrastructure, and automobiles to offer a safe and convenient service for users [1]. Vehicles communicate and exchange information with each other from vehicles and tool booths. Vehicle-to-vehicle (V2V) and vehicle-to-infrastructure (V2I) technologies use the edge infrastructure. V2V communication will exchange information about one vehicle with another, like position, speed, location, etc. [2]. V2I communication enables the transmission of information from a roadside unit to complement V2V communication. V2V and V2I technologies will use dedicated short-range communication to exchange information. Vehicles-to-everything (V2X) technologies are widely used for communication processes such as traffic jams, routing, and accidents [3]. A Transmit Management System (TMS) provides approximate information about the position of the vehicles around the traveller, and it leads to verification of the security of the person. A TMS gives efficient and reliable services to travellers [4]. An Incident Management System is used to identify incidents or accidents that occurred on a person’s travelling route. With this traveller’s aid, it also avoids traffic jams and takes other convenient routes to reach the destination. An emergency management system helps to determine risk and how to avoid risk. This system mostly indicates natural disasters in route [5]. An efficient and secure system was designed for vehicular networks based on the Software-Defined Networking (SDN) paradigm. The proposed architecture ensured improved performance and security for vehicular communication systems, highlighting the role of SDN in providing adaptable, centralized control over dynamic vehicular environments [6]. A review on security, privacy, and decentralized trust management was conducted in vehicular ad hoc networks (VANETs). The challenges of ensuring secure communication and managing privacy were discussed while maintaining trustworthiness in decentralized vehicular networks [7]. A blockchain-based system was proposed to preserve privacy and enable efficient data sharing within intelligent transportation systems [8]. Another blockchain-based solution was investigated for security, privacy, and trust management in vehicular networks. It also examined the potential of blockchain to address security concerns in VANETs, offering decentralized mechanisms to protect users and ensure the reliability of communication [9]. Trust management on the Internet of Vehicles (IoV), encompassing the importance of trust systems in ensuring secure communication and decision-making in vehicular networks, was examined in [10], which identified the key challenges and solutions for building robust trust management frameworks within IoV ecosystems.

The appeal of Intelligent Transportation Systems lies primarily in the advanced services they offer, such as real-time traffic management, improved safety, and efficiency. However, addressing privacy concerns is crucial to ensuring user trust and widespread adoption. Nowadays, both the public and private sectors are providing privacy policies, and this is the main reason for the success of ITSs [11]. The data collected from the users is stored in a database. Differential privacy is applied to protect the floating car data stored and processed in traffic data centres. The main goal is to safeguard traveller data by minimizing the storage of sensitive information wherever feasible and implementing robust security mechanisms to protect necessary data within the database. The approach balances privacy concerns with retaining data essential for ITS functionality and optimization [12]. It focuses on protecting floating car data stored and processed in the central traffic data centre. It helps to identify the traffic conditions and to detect the speed of the vehicles around the travelling road. A laplace mechanism is used to achieve differential privacy [13]. The emergent intelligence (EI) technique is used to analyse, collect, and share information during the privacy process of ITSs [14]. EI is adaptive to complicated and dynamic systems to provide the behaviours for transportation during travelling. Local Differential Privacy (LDP) is another vision of differential privacy that protects travellers’ data from unauthorized parties. It helps users not give unauthorized persons personal information at an appropriate time [15]. There are new difficulties in traffic management, data privacy, and making decisions in real-time brought forth by the advent of autonomous vehicles (AVs), which are fast-changing ITSs.

This study provides a FLPTM system, a framework for optimizing service access and data privacy for AVs, to solve these problems. The FLPTM system uses a Contained Privacy-Preserving Scheme to prevent users’ personal information from being shared and let vehicle train their models on decentralized data. Integral to this architecture is vehicles-to-everything (V2X) communication, which allows for smooth data flow across infrastructure (V2I), pedestrians (V2P), networks (V2N), and vehicles (V2V). C-V2X and the soon-to-be-released 5G-enabled communication systems are examples of modern vehicle-to-element (V2X) technologies beyond Dedicated Short-Range Communication (DSRC). Recent developments have made it easier for AVs to access high-bandwidth data streams in real-time, which is essential for using an FLPTM system in different types of traffic. These communication technologies allow FLPTM to provide strong, scalable traffic management solutions and protect user privacy.

The two most important goals of an FLPTM system’s localized computation are protecting user privacy and enhancing the estimated accuracy of traffic while guaranteeing efficient and secure communication. Improved accuracy and contextual awareness in traffic forecasts are achieved by letting cars train local models with their data. This allows the system to capture region-specific circumstances and patterns. Simultaneously, unnecessary sharing of user data can be protected with centralized servers due to localized processing, which keeps raw data private. This study will ensure that the updated description clarifies its main goal and elaborates on how FLPTM achieves its dual objective of privacy and accuracy. The main contributions of this study are as follows:

- Predictive modelling for AVs using the FLPTM-CPPS system is applied in this paper, which enables traffic models to be trained locally without centralizing data, thus improving the accuracy of traffic predictions.
- FL enables traffic models to be trained locally without sharing raw data and maintains secure communication within the network to ensure data privacy across vehicles and infrastructure.
- A state learning classifier is designed to control service access allocation and user permission revocation by defining different vehicle states and traffic flow states, adapting to real-time conditions to support congestion reduction.
- Vehicle requirements and service access failures optimize communication rates, which are assessed using federated aggregation methods, and efficient predictive management is achieved without compromising data security.
- The efficiency of the proposed approach is evaluated through metrics like access time, adversary impact, response time, and service durability across traffic density and network load conditions.

The CPPS framework focuses on privacy attainment by preventing data leakage via local processing and guaranteeing the secure enforcement of access permissions. Security reinforcement is realized by defending against specific attacks, handling adversarial effects, and maintaining secure state transitions.

Privacy Concerns:

The manuscript focuses on privacy through a Contained Privacy-Preserving Scheme. The main points of this scheme are as follows:

- Local Data Processing: Federated Learning ensures raw data are contained in local devices (vehicles) and not shared with the central servers.
- Privacy Mechanisms: this refers to differential privacy techniques and the privacy-preserving state coalition models that protect user data from unauthorized access.
- State transitions and partial validation ensure privacy against the view of communication failure or adversarial influences.

Security Issues:

The manuscript also contains a few mechanisms for securing data and communication:

- Man-in-the-Middle Protection: the CPPS framework uses authentication protocols, bilinear mapping, and key-based mechanisms to attenuate adversarial threats.
- Access Permission: vehicles must be authenticated and authorized to access communication; this will ensure that access is secured.
- State Modelling for Security: classifier-based state modelling handles potential adversarial effects and enforces security in network interactions.

The rest of the paper is followed by Section 2, describing a recent literature review of the proposed topic. Section 3 details the explanation of the proposed methodology. Section 4 gives the results and discussion. Finally, Section 5 gives the conclusion.

## 2. Related Work

### 2.1. Recent Literature Review

Zhang et al. [16] have introduced FL in an ITS, exposing its capacity to improve privacy and scalability. While tackling new problems, including data imbalance and scarce resources, it investigates FL’s uses in object identification, traffic control, and service delivery. Future trends advocate collaborative alternatives to address these restrictions and guarantee efficient and secure ITS solutions. While the study highlights FL’s potential in improving privacy and scalability, it lacks concrete implementation or testing in real-world ITS environments. Additionally, challenges like communication overheads and data heterogeneity are not fully addressed.

Kaleem et al. [17] improved data privacy, scalability, and real-time decision-making in an urban ITS enabled by the Internet of Things (IoT); this study suggests a customized Federated Learning architecture for Big Data analytics. The design uses an Optimized Federated Averaging Strategy (OFAS) and user-defined learning rates to adjust to changing conditions. The model’s efficacy was demonstrated by testing it on the Udacity self-driving car dataset, where it obtained accuracy levels of over 92% across various node configurations. Although the study demonstrates high accuracy on the Udacity dataset, its applicability to larger, more diverse datasets remains unexplored. The study also assumes ideal network conditions, which may not hold in real-world urban ITS scenarios.

Oladimeji et al. [18] presented the Internet of Things (IoT) linked smart devices for smooth data exchange and communication. This technology improves traffic management, logistics, parking, and safety in transportation. Machine learning, big data, and distributed ledgers have been investigated to enhance smart transportation. The study discusses smart transportation technology and the challenges they face. It covers Wi-Fi, Bluetooth, cellular networks, communication techniques, topologies, and frameworks that allow these applications and systems to function. It examines cloud, edge, and fog computing architectures and frameworks for smart transportation. The study also addresses smart transportation difficulties such as data privacy and security, network scalability, and IoT device compatibility and offers further research. The study provides a comprehensive review but lacks experimental validation of the proposed frameworks and techniques. It also does not adequately address scalability issues in integrating IoT devices across large ITS networks.

Shim [19] presented novel VANET authentication algorithms that conditionally preserve privacy. The study provides malicious but passive KGC attacks; the scheme offers forgery attacks and key recovery attacks. Attacks compromise traceability and unforgeability, allowing anybody to create legitimate signatures on car communications while TA remains untraceable. Furthermore, the attacks demonstrate that by using just one signature, anybody may retrieve the private keys of any vehicle, and using only two signatures, anyone can fake legitimate signatures on any message, all while evading TA’s scrutiny. The writers go into what caused the attacks and how to stop them. The suggested methods are geared toward making VANETs more secure and privacy-preserving. While the study addresses specific vulnerabilities in VANETs, the proposed methods require further testing under various real-world attack scenarios. The approach may also add computational overhead, which has not been analysed.

Kumari et al. [20] presented a CNN–RNN architecture combining FL to forecast real-time smart city traffic. This method achieves a testing accuracy of 99.8% at the number 100 epoch by utilizing CNNs for spatial extraction of features from CCTV images and recurrent neural networks (RNNs) for capturing temporal dynamics. The model’s ability to improve urban mobility in congested metropolitan areas and anticipate at 4.5% demonstrates the ability of AVs to reduce Mean Absolute Error (MAE) with complicated traffic patterns, which beats standard methods such as LSTM, SVM, SAE, and GRU. Despite achieving high accuracy, the study relies heavily on a specific dataset, raising concerns about generalizability. The computational complexity of the CNN–RNN architecture may also pose challenges for real-time deployment in resource-constrained environments.

Munawar et al. [21] addressed concerns about privacy and communication costs related to centralized data approaches, and the work presents an FL approach to passenger demand forecasting in AVs inside smart city transportation systems. Collaboratively training a global model without sharing raw data, AVs use Back Propagation Neural Networks (BPNNs) as local models, boosting privacy. The suggested method outperforms established methods in terms of accuracy and performance metrics (RMSE, MAE, R^2^) when tested on a dataset of 4500 cabs in Bangkok using MATLAB2022b. Additionally, it shows that collaboration improves forecasting accuracy as data availability grows. The proposed FL approach focuses primarily on AV passenger demand forecasting and does not explore its applicability to other ITS functions. Moreover, the communication costs of federated learning in a highly mobile environment are not addressed.

Huang et al. [22] investigated the use of FL and AI in smart transportation, and the study highlights the need for ethical AI to guarantee long-term viability. Responsible AI may foster dispersed intelligence, and the study investigates FL’s role in improving smart transportation systems. To provide smarter, more tailored, safer, and more transparent systems, it also analysed the difficulties of incorporating responsible FL into smart mobility and suggests ways to overcome them. The study emphasizes ethical AI and FL but lacks concrete methodologies or frameworks to implement responsible FL in ITSs. The challenges discussed remain theoretical without experimental validation.

Moulahi et al. [23] integrated the use of FL and Blockchain Technology (FL–BT); this article presented a privacy-preserving method for cyber-threat identification in ITSs and VANETs. While blockchain guarantees secure aggregation of models, FL preserves data privacy. The process includes improving the decision functions of the cars, collecting models using blockchain, and categorizing cyber threats. Although the F1-score and recall were unchanged, the experimental data demonstrated a small decline of 7.1% in accuracy and precision following model aggregation. The approach achieves a harmonious blend of efficiency, security, and privacy in ITS and VANET settings. Integrating FL and blockchain introduces computational overhead, leading to a minor but notable drop in model accuracy. The scalability of this approach in larger ITS networks with diverse datasets is not discussed.

Hu et al. [24] analysed the exchange of model parameters rather than raw data, using FL-assisted traffic demand prediction to overcome data privacy concerns. A Shapley value-based incentive mechanism is used to assess and compensate participating enterprises fairly, and the model uses LSTM and Graphical Convolutional Network (GCN) to capture spatio-temporal patterns. A case study conducted in Hangzhou, China, illustrates how the research idea outperforms conventional models in terms of accuracy in predicting and privacy preservation capacity. While the Shapley value-based incentive mechanism is innovative, its real-world implementation and scalability for large-scale ITS systems remain untested. The study also does not evaluate the impact of communication delays on model performance.

Shen et al. [25] presented that Internet of Things (IoT) edge devices have generated vast amounts of data, making data-sharing privacy a major problem. Evolving privacy preservation learning algorithms for edge computing-based IoT data exchange solve this challenge. The authors apply evolutionary game theory to IoT device-edge node communication and create a reward matrix. Edge nodes constantly alter their techniques to maximize payoffs and block malicious requests to protect IoT data. The ideal evolutionary learning method is derived using a unique algorithm, and the findings are empirically confirmed to verify the IoT data-sharing privacy preservation system is correct. This paradigm prevents malicious infiltration and confidentiality leaks when IoT data are exchanged. The evolutionary learning approach assumes consistent and cooperative behaviour among edge nodes, which may not always be true. The study also does not address the computational demands of its reward matrix optimization in large networks.

Problems like security and privacy issues in Vehicular Ad Hoc Networks (VANETs) must be addressed before deployment. Alshudukhi et al. [26] proposed a lightweight authentication with a conditional privacy-preserving scheme using elliptic curve cryptography to secure communication in VANETs. The scheme combines a Road Side Unit (RSU) and a Tamper-proof device (TPD) to track security issues. The public patterns and the network keys are loaded in the RSU and TPD to avoid further issues. The proposed scheme is cost-efficient when compared with the existing schemes. The lightweight authentication scheme may not fully address scalability in large VANETs with high vehicular density. Furthermore, the dependency on RSU and TPD could introduce single points of failure.

Chougule et al. [27] integrated an ITS with FL-based Privacy-Preserving Asynchronous Training (FL-PPAT) and Vertical Partitioning. The model decreases fuel consumption and idle time at red lights by optimizing the flow of AVs in traffic at level crossings. In addition, it presents a method that considerably improves the passing-vehicle ratio, reaching an acceptable level of 1.33 as opposed to the conventional model’s 0.88. While guaranteeing data privacy among linked vehicles, the suggested method shows improved efficiency in traffic management. The study focuses on specific scenarios like red-light traffic optimization, which may limit its generalizability to other ITS use cases. The impact of communication latency on the performance of the asynchronous training model is not explored.

Huang et al. [28] proposed an approach for privacy-preserving traffic prediction in the IoV: F-STTP-Net, which is based on Federated Learning. It can capture spatiotemporal dependencies without exchanging raw data by subdividing road networks and employing local models with graph attention networks (GAT) and LSTM. The real-world tests demonstrate both the prediction performance and the flexibility across domains. Although F-STTP-Net performs well in capturing spatiotemporal dependencies, its reliance on local models may limit global accuracy in highly dynamic traffic conditions. Scalability across larger road networks remains untested.

Soleimany et al. [29] proposed a multi-level traffic light control system for future smart cities to improve traffic flow, energy efficiency, and air pollution. The system constantly profiles cars, motorcycles, and pedestrians and predicts their movements for better traffic management based on real-time traffic data collected from environmental sensors. Each vehicle and crossroads have localized models that alter the signals depending on current conditions and deadlines for destinations, while a cloud-based model determines the best waiting times. The proposed multi-level traffic light control system may require extensive sensor deployment, which could be cost-prohibitive. The reliance on localized models might also result in suboptimal coordination in highly interconnected urban areas.

Intelligent Transportation Systems traffic management solutions currently have several drawbacks. Since raw data need to be sent to central computers, which increases the likelihood of data breaches, current centralized methods of processing data often violate user privacy. Furthermore, centralized solutions may have difficulty scaling and changing traffic patterns, making real-time traffic forecast and management inefficient. In addition, when dealing with traffic patterns particular to regions or constantly changing, the accuracy of traffic analysis models that rely on static data tends to be poorer. Conventional ITS systems still face serious problems with security, such as being susceptible to hackers. The FLPTM framework, which stands for Federated Learning-based Predictive Traffic Management, makes significant improvements that solve these problems. The suggested architecture guarantees that raw data stay localized to each vehicle by decentralizing data processing using Federated Learning with a Contained Privacy-Preserving Scheme. This method improves traffic forecast accuracy by collecting region-specific trends while enhancing privacy via localized data. To reduce security threats, the FLPTM system incorporates secure communication protocols. It guarantees scalability by allowing distributed processing between vehicles. These enhancements provide an effective, efficient, and secure ITS real-time traffic control method.

### 2.2. Research Gaps

For current ITSs, critical vulnerabilities and inefficiencies include centralized data processing, limited scalability in high-density environments, and inadequate privacy protection from rapidly evolving threats. Many lack mechanisms to mitigate adversarial impacts on service quality and communication reliability. In addition, the dependency on static data precludes real-time decision-making and adaptability to dynamic traffic conditions. Furthermore, high communication costs become an additional limitation for efficiency in real-time applications. The proposed FLPTM framework addresses the above challenges by using Federated Learning for decentralized data processing, a contained privacy-preserving scheme for enhanced data security, and robust adversarial resilience mechanisms to guarantee scalable, efficient, and secure real-time traffic management.

## 3. The Proposed Methodology

**Figure 1:**
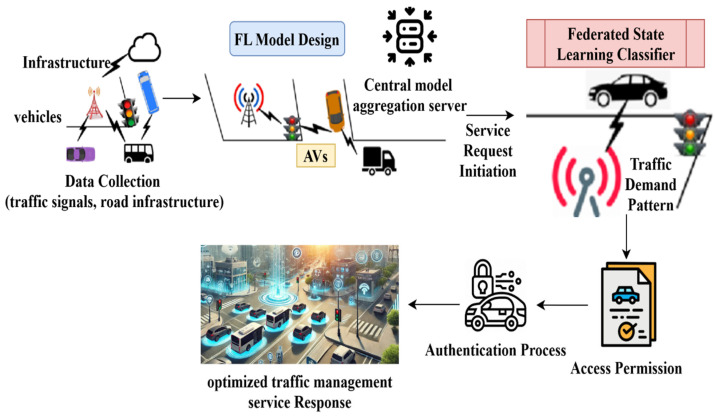
Illustration of the FLPTM system’s working process.

The proposed scheme aims to maximize the vehicle’s service endurance by reducing the adversary’s impact on the mobile environment. The adversary considered in this scheme is the man-in-the-middle that interrupts the services between vehicles and service providers. In the service allocation process, the vehicle’s state is retained if the allocated service sustains regardless of the adversary density. The proposed scheme’s functions are illustrated in Figure 1 for ease of understanding.

The vehicles are interconnected through access points and other infrastructure units. Therefore, V2V and V2X communications are familiar with the proposed scenario. A federated state learning classifier analyses the traffic demand patterns, classifying the vehicles based on their security parameters. The functions are classified as state modelling and service processes. Beyond state modelling, service procedures facilitate a wide range of operational and communication activities within the ITS, making them indispensable. Service processes oversee the system’s many components, including infrastructure, cars, traffic management systems, data interchange, coordination, decision-making, and state modelling. They ensure everything is in sync and communicating well, allowing route optimization, real-time updates, and dynamic traffic management. In addition to assuring data privacy and security during transmission, resolving exceptions and keeping the system reliable are also responsibilities of service processes. Service processes improve the system’s responsiveness, scalability, and overall performance by providing these functions, which allow it to respond to changing user demands and traffic circumstances. In the state modelling, access permission and authentication are administered. By initiating a service request from the AVs, a vehicle verifies its access permissions before beginning the communication, thereby going through an authentication process whenever it asks for service. After the vehicle has been verified, it is permitted to join the network for efficient traffic control. The optimized and accurate traffic management service response prediction yields the system’s real-time responses to traffic flow and communication; these changes improve network security and efficiency. The classifier learning process defines the states and functions. On the contrary, requests and responses are performed in the other process. Vehicles and infrastructure (as shown by symbols like automobiles, buses, and traffic lights) initially gather data from road infrastructure and traffic signals. Next, the data are processed locally, and only model updates are sent to the central Federated Learning (FL) server for aggregation. This design and training process represents a decentralized machine-learning model for traffic management.

In this model, each infrastructure node and vehicle participate in the training process without sharing raw data. A global model is created by combining the local models from all dispersed nodes (infrastructure and vehicles) at the central model aggregation server. After combining the models, the system is signalled to evaluate the traffic demand pattern in light of the present traffic conditions by initiating a service request. The federated state learning classifier is used to assess and classify the traffic demand pattern to make the best decisions possible about traffic management. After traffic demand classification, the system uses authentication to guarantee that AVs have secure access to traffic management services. After authentication is successful, access authorization is given, enabling the autonomous vehicles to obtain optimum replies for traffic management. These responses serve as the final outputs, guaranteeing that autonomous vehicles can navigate traffic efficiently.

The proposed framework starts by initializing a list of vehicles, infrastructure, and service request states, followed by the training of local models on vehicle data $\rho_{S}$ and infrastructure data $\rho_{I}$. The parameters $\rho_{I}$ and $\rho_{S}$ denote the availability of the infrastructure and services, each ranging from 0 to 1. Values closer to 0 are less available, and closer to 1 are more available. This continuous scale models partial availability, capturing scenarios like degraded performance or intermittent connectivity, which cannot be represented by binary states (e.g., true/false). These local models are then sent to the system for aggregation into a global model, which is distributed back to all autonomous vehicles (AVs). The system decides on the service type based on conditions such as high-security requirements (when both $\rho_{I}$ and $\rho_{S}$ are 1) high traffic demand or regular service needs. Each vehicle’s service access is updated accordingly. Privacy is key, and service access is revoked if a vehicle no longer meets the required privacy standards. The system predicts traffic parameters and conditions (T) and evaluates performance using metrics related to service effectiveness. τ denotes the time delay between a vehicle requesting a service and the time its response is received. This value is essential for assessing the system’s communication efficiency. The implemented FLPTM system for ITSs is an Algorithm that outlines the main components, including input, output, and the decision-making process regarding service access based on vehicle states and adversary impacts. The function takes a list of vehicle requests, infrastructure, and service provider availability and outputs a list of service grant statuses for each request. The function iterates through each vehicle request and checks the availability of resources and service providers to determine whether to grant, deny, or put the request in a pending state. The Algorithm 1 provides a simplified view of the decision-making process in the proposed system as:
**Algorithm 1:** Proposed FLPTM system for ITSs.INPUT: vehicle data $\left(\rho_{I},\rho_{S},servicerequest,trafficdemand\right)$ OUTPUT: service response Initialize list of V,S,I
for each vehicle, V do   train a local model using $V^{\prime }$s data  send a local model update    while the system is running, do     local models = gather all local model updates       aggregate (local models)       send an updated global model to all AVs      if $\rho_{I}=1$ and $\rho_{S}=1$ then    service = high_security    else if traffic demand is high, then    service=traffic optimization   else   service = regular service update service access for V with S for each vehicle, V do    if the vehicle state no longer meets privacy, then
    G=0    revoke service access for vehicle V   else
    G=1   Predict τ and T end for evaluate performance evaluation using metrcis

### 3.1. Adversary Impact Representation

**Figure 2:**
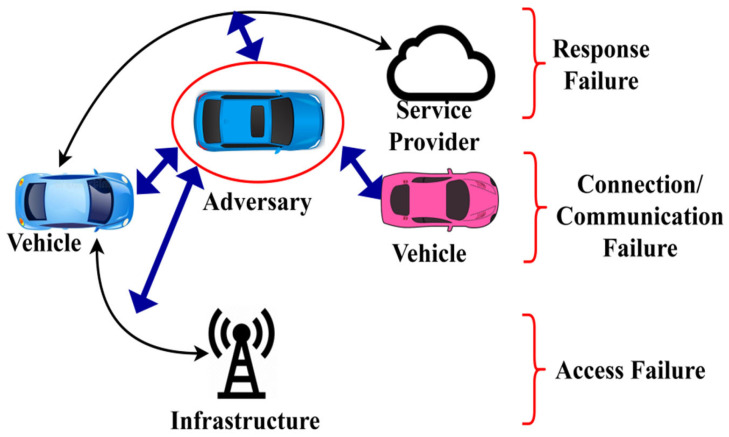
Adversary impact representation.

The man-in-the-middle adversary model is considered in the function validation. Figure 2 illustrates a schematic representation of the same. A man-in-the-middle intruder causes response, communication, connectivity, and access failures. The impact of a man-in-the-middle attack, including response delays, communication disruptions, connectivity issues, and access failures, depends on the adversary’s position within the network and the density of adversarial nodes in the ITS scenario. The proposed scheme must confront the abovementioned issues without degrading the communication performance.

First, the permission grant and service access are defined for a vehicle using Equations (1a) and (1c), respectively:

(1a) $$\forall V,R\in t,G=\left\{\begin{array}{c} 1,\;\mathrm{if}\;\rho_{I}.\rho_{S}=1\\ 0,\;\mathrm{if}\;\rho_{I}=0\;\mathrm{or}\;\rho_{S}=0 \end{array}\right.$$

such that

(1b) $$p\;dt=\sum_{i=1}^{t}\frac{∆R}{R}=1$$

and

(1c) $$p\left(t\right)=\left\{\begin{array}{c} G.\mathrm{pdt}-\frac{\tau \rho_{I}}{\rho_{S}},\;\mathrm{if}\;\rho_{I}<\rho_{S}\\ G.\;\mathrm{Pdt}-\frac{\rho_{s}}{\rho_{I}},\;\mathrm{if}\;\rho_{S}<\rho_{I} \end{array}\right.$$

In Equation (1a), the variables V,R, and t represent vehicles, requests, and time. For a response (∆R), the available service providers (s) respond if the infrastructure and S are available. The term R∈t says that the request R is relevant to, or belongs to, some time interval t. The statement establishes a temporal relationship that the request R is valid or relevant during the time t.p(t) represents the probability that a service request is valid or relevant at a predetermined time interval t. Similarly, “p dt” is the probability that a valid service request will occur in the infinitesimal time interval dt, in consideration of system constraints like infrastructural and service availability. In the Equation (1b), if the summation uses i as the index, but if the element ∆R/R does not depend on i, then ∆R/R is a constant concerning i. In that case, the summation multiplies the constant ∆R/R by the number of terms in the summation, i.e., $\;\sum_{i=1}^{t}\frac{∆R}{R}=n\times \frac{∆R}{R}$.

In Equation (1c), G⋅p dt is the instantaneous probability density of granting access, where p is a probability density function describing instantaneous system conditions, for example, resource availability or security check over an infinitesimally small interval in time dt. G⋅P dt: Here, G now represents an accumulated probability of granting access and P becomes the accumulated probability given for more general system behaviour, accounting for a time-averaged fashion or threshold satisfaction, again scaled by dt.

The availability of infrastructure and service providers is defined as $\rho_{I}$ and $\rho_{S}$. The grant process is defined as G, and the permission P concerning ∆R and failure probability (τ) is formulated in any instance and is retained at a high level. The failure probability (τ) is deduced from the cumulative time delays. It expresses the probability that a service request will not be executed within the acceptable temporal thresholds, thus expressing the system degradation. This increases the service’s endurance by reducing errors. The permission grants for G=1 and 0 are independently considered for defining a state. First, the state is defined as ∀V and R, the G=1, and hence $\rho_{I}.\rho_{I}=1$. The proposed scheme defines three states: grant, deny, and pending. The grant state ensures service distribution to the V, enduring its span while preserving its privacy through FL mechanisms. The denied state halts the service distribution due to privacy violations and adversary impact. Contrarily, the pending state defines the actual vehicle’s involvement in service sharing. This means it possesses the states of either grant or deny. If a grant occurs, it augments the service endurance; a denial increases service failures. Initially, the service level for a vehicle is defined as in Equation (2).

(2) $$\left.\begin{array}{c} \hat{S}=\frac{\rho_{I}}{\rho_{S}}+\left(1-\frac{∆R}{R}\right)+\prod_{i=1}^{t}P\;dt-\tau_{i}\\ where\;\tau =(R-∆R) \end{array}\right\}$$

In Equation (2), τ (failure probability) is computed as $\tau =\left(R-∆R\right),$ where R  denotes the timestamp when a service request is made, and ∆R represents the timestamp when the response is delivered. This metric is crucial in determining the traffic management system’s responsiveness and identifying service provision bottlenecks. The service level $\hat{S}$ defines the flexibility provided to vehicle V throughout i=1 to t such that G=1.If G = 0, then τ>ρ di ∀i ∈[1t], and the service failure is accounted for. The decision as to whether the system is within acceptable performance limits is determined by the comparison between τ and ρ, where ρ is the fraction of system resources available to serve requests. If  τ>ρ, it would imply the time delay is greater than the capacity of the system to handle requests; therefore, due to this fact, system performance is degraded or results in service failure. Insights like this are instrumental in pre-emptive traffic management for autonomous vehicle reliability.

### 3.2. Privacy Preserving State Coalition Model

**Figure 3:**
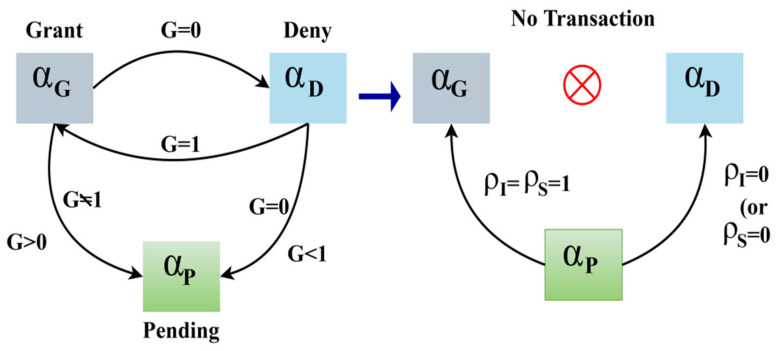
State coalition representation.

A state coalition paradigm is provided for FL environments where grant $\alpha_{G}$, deny $\alpha_{D}$, pending $\alpha_{p}$ service states are securely represented. Each transaction between states is verified using FL updates, guaranteeing that transitions retain data privacy and secure accessibility across distant entities. Based on $\hat{S}$, the service grant state of a V is defined as $\left\{\alpha_{G},\alpha_{D},\alpha_{p}\right\}$ where the grant, deny, and pending are represented. A common coalition between the states is represented in Figure 3.

In the state coalition, granting to deny and vice versa rely on G alone. Whereas $\alpha_{p}-\alpha_{G}$ and $\alpha_{P}\;\mathrm{to}\;\alpha_{D}$ transactions are decided based on $\rho_{I}$ and $\rho_{S}$. Therefore, the occurrence due to vehicle movement and handoffs in different $\rho_{I}=1$ instances requires the above intermediate transactions. The transaction between $\alpha_{p}$ and $\alpha_{D}$, and $\alpha_{P}$ and $\alpha_{G}$ are defined using Equation (3)

(3) $$\left.\begin{array}{c} \prod_{P-D}=\rho \left[\left.P\left(t\right)+\frac{\rho_{S}}{\rho_{I}}1-P(t)\right|\hat{S},t\times G\right]\\ \left[\prod_{P-G}=\rho G+\frac{∆R}{R}\left(1-\rho_{S}\right)\left|\hat{S}\right.,\left(1-\rho_{I}\right)\right] \end{array}\right\}$$

In Equation (3), the variables $\prod_{P-D}and\prod_{P-G}$ denote the transaction for the appropriate states. This is connected with $\hat{S}$ when the service is sustained; hence, the access and connected failures are reduced. The state models for transactions are used to provide different authentication formats. It depends on the state and action as defined in $\rho_{I}=1$ or $\rho_{S}=1$. Contrarily, if $\rho_{I}=\rho_{S}=1$, then the ∆R is high, reducing τ; the alternate case is the privacy-preserving. If a transaction $\prod_{P-D}$ is observed, then partial transaction authentication is required. Contrarily, if $\prod_{P-G}$ is observed, then a complete authentication sequence including V and S is required. The first preserves the V, disconnecting τ induced failures, whereas the later part requires V and S authentication, preserving service endurance. The authentication for $\prod_{P-D}$ is discussed as follows. In this process, a conventional bilinear mapping-key-based authentication is used. For a service grant process where $\rho_{S}=\rho_{I}=1$, the bilinear pairing between V and I is defined as $\left(B\times B\right):\to [A_{prim},S_{prim}{]}^{G}={\left[A_{prim}\right]}^{G}$. The bilinear pairing formulation is relevant to the vehicle and service provider, as it ensures their identities and the associated privacy primitives are securely linked and protected within the service grant process. Here the $A_{prim}$ and $S_{prim}$ refer to the vehicle’s and service providers’ primitives for privacy. The primitives include a non-replicated key $\left(k\right)$, a random generator α, and $\hat{S}$. Therefore the $A_{prim}$ and $S_{prim}$ are defined as in Equations (4a) and (4b), respectively, ∀ G∈t,

(4a) $$\begin{array}{c} A_{prim}=[V,(G,K)\left\|\left(\hat{S}.\alpha \right)⨁B^{\alpha }]\right. \end{array}$$

(4b) $$S_{prim}=\left[S,K\frac{\left\|\alpha \right\|}{V},\rho_{s}⨁\frac{1}{B}\right]$$

(4c) $$Provided\left\{\left(G,K\right)\left\|\hat{S}.\alpha \right\|.\rho_{s}⨁\frac{1}{B}\right\}={\left(G.k\right)}^{\alpha }.{\left(\frac{1}{B}\right)}^{\alpha }$$

The “provided” condition given in Equation (4c) is the congruency in verifying the privacy between V and I, and (I,S). If the congruency is retained, then the state is retained as $\prod_{P-D}$ false $\prod_{P-G}$ is observed.

### 3.3. Integrated FL into the Authentication Process

**Figure 4:**
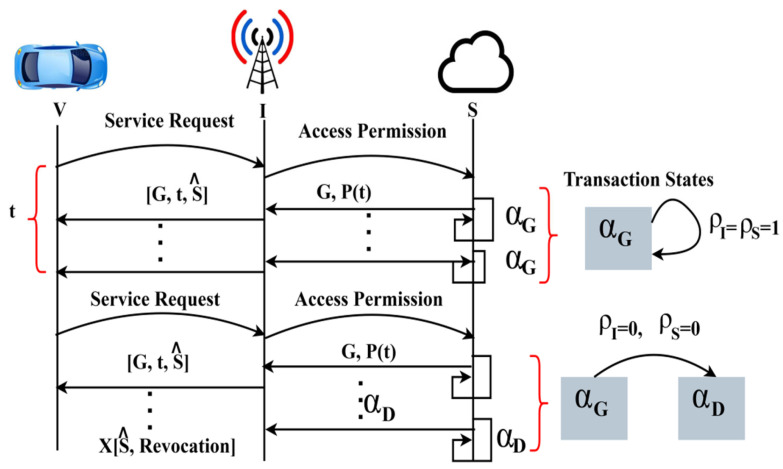
Privacy-preserving authentication.

The congruence-based privacy preserving between V, I, and S is presented in Figure 4. This illustration is observed before a privacy breach/communication failure occurs.

The above Figure presents the validation between different transaction states wherein $\rho_{I}=\rho_{S}=1$ or 0 is considered. There are two possibilities for providing authentication and privacy preservation: $\prod_{G-G}$ (i.e.,) $\alpha_{G}$ is alone true and $\alpha_{G}$ to $\alpha_{D}$ is experienced. In the first case, complete privacy will be retained for the V and services. As discussed earlier, the v′s privacy and authentication are expected alone in the second transaction. Therefore, by pursuing Equations (4a) and (4b), the primitives are exploited to maximize the communication rate. In Lakes privacy experiences, the primitives (of V) are revoked, suspending it from the I connection. Thus, the changes are reverted using the states, and in a reconnection, the $\prod_{P-D}$ or $\prod_{P-G}$ is considered. Therefore, the first authentication covering V,I, and S is given by Equations (5a)–(5d).

(5a) $$\left[A_{prim},S_{prim}\right]=\left[\frac{∆R}{R},\left\|\hat{S}\right\|,K\right]⨁[B{]}^{\alpha }$$

(5b) $$\left[A_{prim}\left(t\right)\right]=\left(G,K\right)\left\|\hat{S}\right\|.{\left[\frac{1}{B}\right]}^{\alpha }.P\;dt$$

(5c) $$\left[S_{prim}\left(t\right)\right]={\left[\frac{1}{B}\right]}^{\alpha }.K⨁\hat{S}$$

(5d) $$\prod_{G-G}=\left[P\;dt.\frac{∆R}{R},1\right],\forall \left(1,t\right)$$

In Equation (6), the modifications are pursued between SandI, and hence, privacy is retained for $\hat{S}$. This ensures intruders have less access to the services at a high communication rate. Therefore, the privacy between V,I, and I is high, and the service access is restored. Contrarily, state transaction is retained in $\alpha_{G}$ such that ${⨅}_{G-G}$ is used for verifying t. In the other authentication, partial privacy is ensured, wherein ${⨅}_{G-P}$ is induced. The process illustrated in Figure 4, i.e., $\rho_{I}=\rho_{S}=0$, represents the failure in t; therefore, an adversary impact is experienced. Therefore, the partial privacy requirements are retained based on the previous state. If the previous state is $\alpha_{n}$, then new validation and authentication are initiated. If the previous state is $\alpha_{p}$, then the state of the vehicle is either grant $\alpha_{G}$ or deny $\alpha_{D}$. Therefore, partial privacy (for V alone) is retained. In this scenario, the privacy is preserved based on $\prod_{G-G}$ and from this, if the V requires authentication, it performs $A_{prim}$ and $S_{prim}$ exchange. This is induced in t∀ authentication, concealing the communication. This partial privacy is ensured in $\prod_{P-D}$ and $\prod_{G-P}$ transactions. The process is defined using Equations (6a)–(6c) for both transactions.

(6a) $$\left[A_{prim},S_{prim}\right]=\left(1-\rho_{S}\right)\left(1-\rho_{I}\right)⨁(G,K)$$

(6b) $$A_{prim}\forall P\left(t\right)=\left\{\left(1-\rho_{I}\right)⨁G\left\|K\right\|\right\}\frac{\hat{S}}{t}$$

(6c) $$S_{prim}\forall P\left(t\right)=\left(1-\rho_{S}\right)⊕{\left[\frac{1}{B}\right]}^{\alpha }.\hat{S}$$

Validate

(6d) $$A_{prim}\forall P\left(t\right)=S_{prim}\forall \;P\left(t+1\right)or\;P\;dt$$

The above validation given in Equation (6d) ensures the $\rho_{I}=1$ or 0, whereas $\rho_{S}=1$ or 0 need not be verified. This reduces the communication cost provided for V2V and V2I information exchange. The above is valid until $\prod_{P-P}$ or $\prod_{D-D}$ is not achieved in any t. Hence, the communication rate is expected to be high in the abovementioned case. The contrary part requires a proper classification of a revoked/persisting V in the communication scenario. Here, a V’s revocation does not require the above authentication, reducing the communication cost. It depends on $\alpha_{G}$ to $\alpha_{D}$ transactions to provide a denial of service access. First, the $\rho_{S}$ is verified and proceeded by $\rho_{I}$ requirement, and hence revocation with the last known $\hat{S}$ is achieved. The process verifies the current and previous state is expected to be in $\alpha_{G}$ for new communication. The transaction under different $\rho_{I}=0$ or 1 and $\rho_{S}=0$ or 1 is defined as in Equation (7).

(7) $$\left.\left.{⨅}_{P-D}=\frac{\rho_{I}}{\rho_{S}}\left(1-\tau \right)+\frac{∆R}{R}\right|{⨅}_{P-G}=\left(1-\tau \right).G\left(\frac{1}{R}\right)\right\}$$

The chance that leads to modification in different t is evaluated using Equations (3) and (7). In Equation (7), $\hat{S}$ is not accounted for as the service level is unknown (unavailable) in $\alpha_{P}$ state.

### 3.4. Different State Transactions

**Figure 5:**
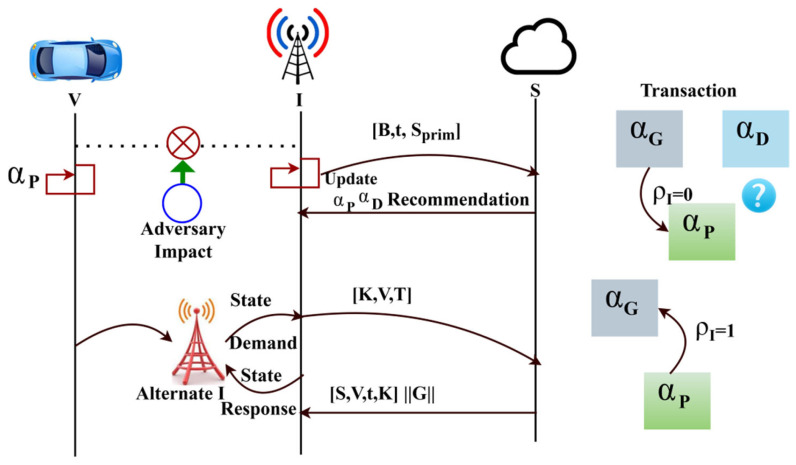
Different state transactions.

The process for different state transactions based on V-to-S communication is illustrated in Figure 5.

As in the above illustration, $\alpha_{P}$ to $\alpha_{G}$ is verified through a new I, and the previous state demand and response are required here. The state change is observed for any t with precise k for G=1. Therefore, the $\rho_{I}=1$ is retained within ${⨅}_{P-G}$ transaction. Therefore, a vehicle revocation case is not required. Contrarily, the discrepancy due to B and τ by equating Equations (3) and (7) induces a revocation. Therefore,

(8a) $$\left.\begin{array}{c} \rho \left[P\left(t\right)+\frac{\rho_{S}}{\rho_{I}}\left\{1-P\left(t\right)\right\}\right]=\frac{\rho_{I}}{\rho_{S}}\left(1-\tau \right)\frac{∆R}{R}\forall {⨅}_{P-D}\\ and\\ \rho \left[G+\frac{∆R}{R}\left(1-\rho_{S}\right)\right]=\left(1-\tau \right).G\left(\frac{1}{R}\right)\forall {⨅}_{P-G} \end{array}\right\}Same\;State$$

(8b) $$\left.\begin{array}{c} \rho \left[P\left(t\right)\right]=\frac{∆R}{R}\left[as\;\rho_{I}=\rho_{S}=1\;and\;hence\;\tau =0\right]\\ \begin{array}{c} and\\ t=1-\left[\frac{\rho \left(G\right)}{G}.R\right]\left[as{\;\rho }_{I}=\rho_{S}=1\;but{\;\rho }_{S}=0\right] \end{array} \end{array}\right\}Transaction$$

In the above Equations (8a) and (8b), two different constraints are balanced:G and $\left(\rho_{I},\rho_{S}\right)$. If both constraints are satisfied, the same state is retained; otherwise, a transaction is required. This transaction ensures the revocation is the same across different intervals. Therefore, τ≠0 whereas τ=0 to τ≠0 has to be verified in different t. Thus, the V is suspended from the communication due to adversary impact. If the adversary impact is overcome, the validation pursues a partial validation, preventing the impact over V. Therefore, the revocation denies S access for multiple t and persists to be the same, preventing different verification and privacy patterns. The FLPTM system handles state transitions with high sensitivity to the current network state, such that when an adversarial impact is lessened, partial V revalidation is performed for secure communication.

### 3.5. User Revocation

The proposed FLPTM framework includes mechanisms for the user revocation process to distinguish a change in service access and vehicle state transactions. In the revocation process, the constraints in Equations (8a) and (8b) are validated, whereas Equation (1) with $\rho_{I}=1$ or $\rho_{S}=1$ is modified. Hence, in this case, the change is performed with an augmentation in multiple t. These mechanisms identify, isolate, and revoke access for compromised entities, helping to maintain security and continuity without centralized intervention.

However, this occurs in different t, and therefore, adversary impact is reduced. The $V^{\prime }s$ state is retained in the previous transaction, preventing privacy leakage. For a new vehicle request, the permission is denied at the same interval, and a persisting vehicle’s permissions/access from the current t are revoked. The revoked process is defined by Equations (9a)–(9c).

$\forall \rho_{S}=0,$

(9a) $${⨅}_{P-G}=\left(1-\frac{\tau }{R}\right)*\left(\hat{S}-\frac{\tau }{V}\right)$$

(9b) $$\begin{array}{c} such\;that\\ \left(1-\tau \right)\frac{G}{R}=\left(1-\frac{\tau }{R}\right)\times \left(\hat{S}-\frac{\tau }{V}\right)\;[Equation\;\left(7\right)\;with\;above]\\ \begin{array}{c} \left(1-\tau \right)G=\left(R-\tau \right)\left(\hat{S}-\frac{\tau }{V}\right) \end{array} \end{array}$$

(9c) $$\left.\begin{array}{c} \mathrm{if}\;R\;;>\;0\;:\;G\;=\left(\frac{R-\tau }{1-\tau }\right)*\left(\hat{S}-\frac{\tau }{V}\right)\\ \mathrm{else}\;\mathrm{G = R}\;*\left(\hat{S}-\frac{1}{V}\right) \end{array}\right\}$$

The grant is defined in the above Equation for R requests, and if a V retains its state in $\alpha_{D}$, then R=0, and hence G=0. This means the V is revoked from $\rho_{I}$ and $\rho_{S}$, deviating service access. On the other hand, revoked users are analysed for their liability, and hence the authentication follows $A_{prim},S_{prim}$. The parameter $\hat{S}$ represents the predicted and calculated state of the vehicle, which is related to privacy conditions in access control. If a V meets the required conditions (based on privacy, traffic, and security factors), it is granted service access and G=1. The AV’s state transition is influenced by the predicted service allocation, the vehicle’s traffic behaviour, and the communication rate. In Equation (4), the partial authentication is induced to preserve a $V^{\prime }s$ privacy regardless of $\rho_{I}=1$ or $\rho_{S}=1$. Pursued by this, the revoked user is allocated a service until the condition (transaction) in Equation (8) is achieved. This defines a new $\hat{S}$ for the user/vehicles in the communicating scenario. In Table 1, the G for different “t” is presented.

The G observed at an average for different “t” is presented in Table 1. This is based on ${⨅}_{P-D}$ observed in the different states available. The service endurance is maximized if ${⨅}_{P-G}$ is high, provided $\rho_{s}.\rho_{I}=1$ and the constraint in Equation (8) is satisfied. Contrarily, the G requires ${⨅}_{P-D}$ and $\hat{S}$ for providing flawless dissemination. The above factors reduce the adversary impacts, containing multiple non-feasible factors in “t”. Table 2 presents the service endurance and communication cost for different vehicle densities.

**Figure 6:**
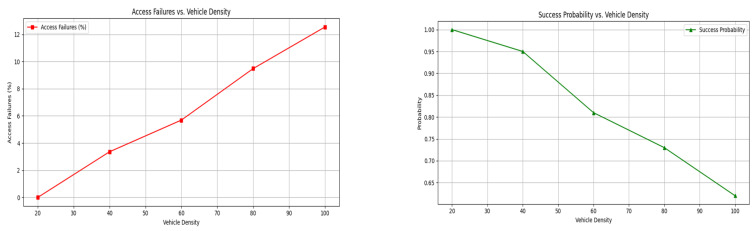
Service endurance and access failure analysis with different vehicle densities.

An analysis of service failure, communication cost, and service endurance is presented in Table 2. The endurance is retained based on the G factor defined in two equations. The communication cost increases if G is high; hence, the service failure rate is lower. These two factors under ${⨅}_{P-G}$ and $\rho_{S}.\rho_{I}=1$ maximizes service endurance without increasing the communication cost. Figure 6 presents the service endurance and access failure percentage analysis with different vehicle densities. The probability refers to the probability of a successful service request given certain conditions of vehicle density.

**Figure 7:**
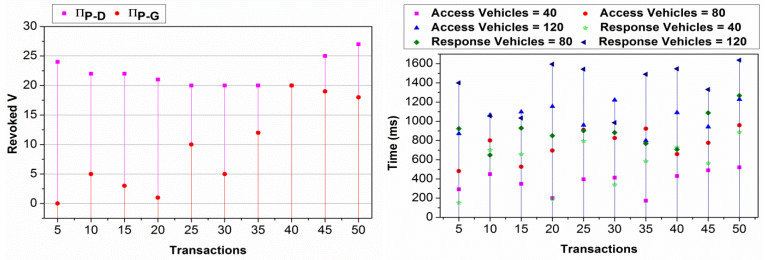
Revoked V and time analysis for transactions.

In scenarios where the probability of a successful service request equals 1, failures in access should theoretically not exist. However, observed failures in access can still be attributed to transient network conditions that might cause delays in authentication or temporary unavailability of infrastructure. These are independent factors from the intrinsic success probability of the service request itself. The service endurance analysed using the FLPTM system represents how long the vehicle continues to have access to the service before conditions change. The access failure could occur when the system denies service to a vehicle due to high traffic demand and privacy concerns. The probability considered is $\rho_{I}.\rho_{S}=1$ wherein the individual ratios may vary. As the endurance increases, access failure decreases confined to the $\hat{S}$. In the maximizing probability, the V determines the available “t”, so a process is defaced. Therefore, the lower the vehicle density, the higher the endurance and the less the failure. The independent and joint state definitions and ${⨅}_{P-G}$ determinations reduce the failure in resource access. The proposed scheme balances V and G for different privacy constraints that maximize performance. Figure 7 presents the revoked V, access, and response time for different transactions and vehicles.

In Figure 7, the $V^{\prime }s$ revoked and the time for different transactions are analysed. The $V^{\prime }s$ revoked are analysed under ${⨅}_{P-D}$ and ${⨅}_{P-G}$ transactions. In ${⨅}_{P-D}$ the revocation is high as ${⨅}_{G-P}$ is achieved first; hence, the vehicle is not included in the communication. Contrarily, ${⨅}_{P-G}$ reduces the revocation as both $\alpha_{D}$ vehicles and new ones are augmented for communication. This requires different access and response times, controlling privacy, and $\hat{S}$. The changes are predominant in providing access to the S, and $\rho_{s}.\rho_{I}=1$ is retained. Therefore, the access is mapped to the S based on their incoming time and the response. In different ${⨅}_{P-G},{⨅}_{P-D}\times {⨅}_{G-P}$, access, and response are provided at precise intervals.

## 4. Results and Discussion

### 4.1. Data and Comparative Study

This section analyses the proposed scheme’s performance using comparative analysis. The experiment is modelled using vehicular SIM, considering 130 vehicles distributed on a highway with three intersections. A vehicle is allocated a maximum of nine instances for service-sharing augmentation. Three vehicle states and 50 transactions are considered to identify the performance of access time, adversary impact, response time, service endurance, and communication cost. The methods OFAS [17], BPNN [21], and FL–BT [23] are accounted for in the comparative analysis with the proposed FLPTM system for ITSs.

### 4.2. Access Time

**Figure 8:**
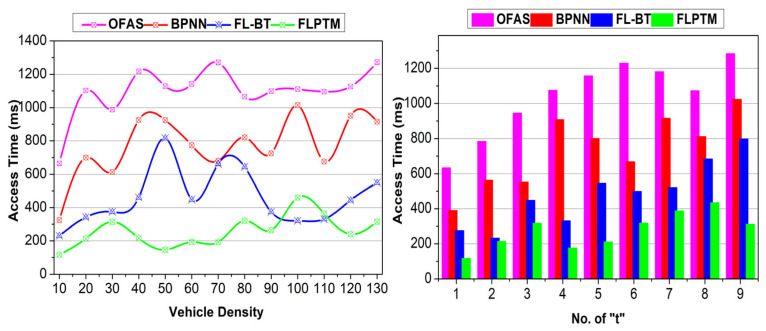
Access time comparisons.

Figure 8 presents the comparative analysis for access time for different vehicle densities and “t”. The FLPTM framework emphasizes decentralized processing, allowing vehicles to handle requests locally without relying on a central server. Integrating vehicle requests based on transactions aligns with the FLPTM system’s research goal to enable vehicles to operate autonomously.

The access time is comparatively less for different t and V by maximizing the request process rate. In the proposed scheme, the $V^{\prime }s$ are integrated based on transactions defined by $\alpha_{D}$ and $\alpha_{P}$. The pending state provides additional delay for the R in different t. First, if $\alpha_{p}$ tends to $\alpha_{G}$, then G=1 is acquired, and hence, access time is less. Contrarily, if τ≠0 is observed, the partial privacy-preserving feature is instigated to maximize access. The $\hat{S}$ is retained for the previous case, whereas the $\hat{S}$ is defined from 1 for the second case. In G, assessment based on ${⨅}_{P-G}$ balancing as in Equations (9) and (8), the $\rho_{S}=0$ or $\rho_{I}=1$ is first attained. If $\rho_{S}=1$ is achieved, then τ tends to 0; hence, the revocation is denied. Therefore, access to service is provided instantaneously without reducing ∆R. In addition, the state learning-based allocations reduce the adversary impact and frequent disconnections. This turns out in ${⨅}_{P-G}$ and ${⨅}_{G-G}$ independently. Therefore, the v′s requests are momentarily analyzed without additional communication costs. The split in $\rho \left[P\left(t\right)\right]$ and τ, as in Equation (8), defines the access level without intersection. Hence, incorporating the above features, the proposed scheme reduces access time.

### 4.3. Adversary Impact

**Figure 9:**
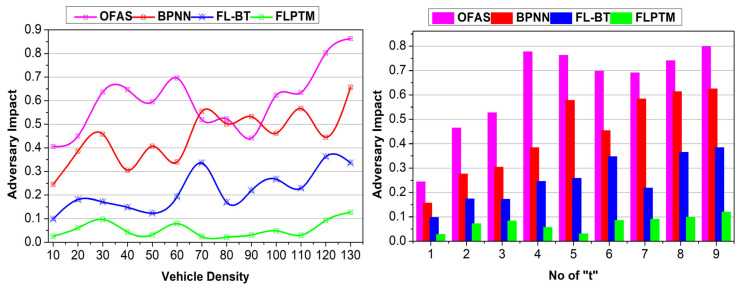
Adversary impact comparisons.

The proposed scheme achieves less adversary impact compared to the other methods. An illustration of the same is presented in Figure 9 for different v and “t”. The considered impact of the man-in-the-middle adversary is combated using transactions and state modelling. First, the G for a V is designated as 1, such that $\rho_{I}.\rho_{S}=1$ is satisfied.

Two cases of adversaries are considered, i.e., the adversary’s location is to be considered. In ${⨅}_{P-D}$ and ${⊓}_{P-G}$, the states are retained, and new identity-based privacy features are retained. Therefore, regardless of the adversary’s density and location, the transaction defines its impact. For $\hat{S}$ defined in multiple instances of ${⊓}_{P-D}$ and ${⊓}_{P-G}$, $\rho_{s}=\rho_{S}=1$ is verified. Based on this condition, validation $\left(P_{s}⨁\frac{1}{B}\right)$ ensures secure communication between the $V^{\prime }s$. Therefore, a “t” that breaks the closure reduces the adversary’s impact. In this context, the V is suspended from I, and hence $\rho_{I}=0$. This means the least possible chance of $V^{\prime }s$ privacy being lost is ensured. Further privacy post-transaction verification maximizes high security, reducing the adversary impact.

### 4.4. Response Time

**Figure 10:**
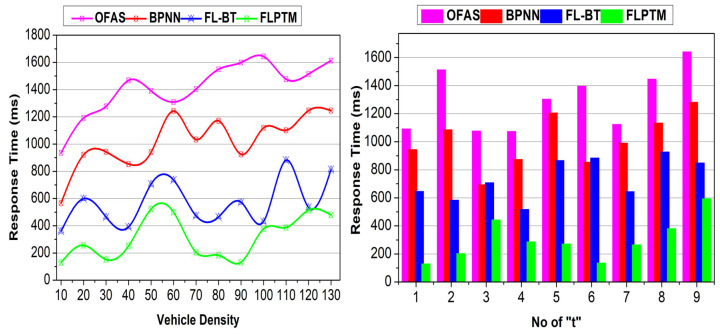
Response time comparisons.

FL’s resilience is achieved through a decentralized approach in which each AV performs local assessments $\rho_{I}$ that provide secure features $\left[G,t,\hat{S}\right]$ in $A_{prim}$ for preserving “t”. FL allows AVs to train on their data without sharing raw data, allowing them to preserve local models. This improves privacy and reduces the computed overhead. Using FL, the proposed approach ensures that vehicles and the central server S establish separate, secure communication channels, allowing ongoing network communication sessions. The proposed scheme achieves less response time than the other methods (Figure 10). The access is concurrent and swift for different V under contained privacy. In the permission delegation, $\rho_{I}$ and $\rho_{S}$ constraints are satisfied for maximizing ∆R. However, if an adversary impact is observed, the transaction determines the V state. Here, $\frac{∆R}{R}$ is the reward factor that maximizes the communication rate without compromising time. The independent/joint authentication for V and session “t” is administered in the privacy retaining case. Therefore, G=1 and, hence, service response is high. For the R in “t”, the ∆R is congruent at some far “t”; therefore, response time is less. In cases where ∆R fails to be met, the applied local privacy protocols prevent the next upcoming session breakdowns and mitigate the risk of adversarial interference, ensuring continuity during FL sessions. On the other hand, an independent privacy-retaining vehicle does not need to ensure false communication. This is confirmed based on $\hat{S}$ and the final validation is performed based on $\left(A_{prim},S_{prim}\right)$. It provides durable communication security, preventing communication τ. Therefore, the passive communication support and interruption in V2V or V2X is less in the proposed scheme, requiring less response time.

### 4.5. Service Endurance

**Figure 11:**
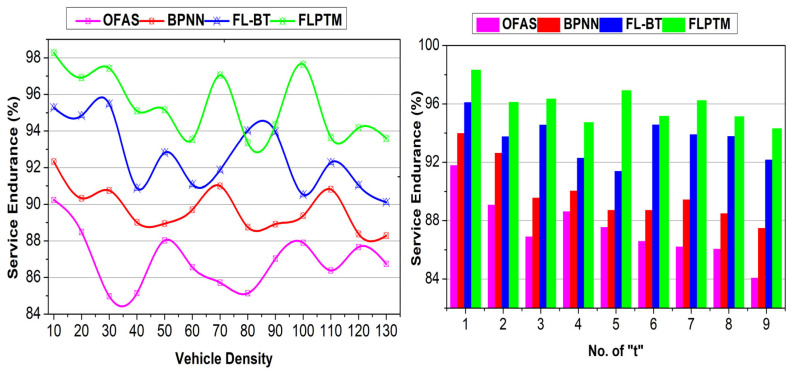
Service Endurance Comparisons.

FL enables local privacy assessments, improving the ratio $\frac{∆R}{R}$ to prevent communication overheads. Each $V^{\prime }s$ local updates are only transmitted when significant model improvements are detected; ${\rho_{I}-\rho }_{S}=1$ guarantees an optimal use of communication channels. Additionally, $A_{prim}$ enables reliable communications when $\rho_{I}=0$, meaning only minimal control data are required between $V^{\prime }$ s and the central node I. Then, state identification through local training allows Vs to monitor their communication needs autonomously, reducing the frequency of global model updates. For instance, Equation (7) highlights the balancing mechanism used to maintain performance while optimizing $\frac{∆R}{R}$ constraints can be validated across AVs and prevent adversarial effects. The proposed scheme retains the communication session without additional computation/overhead. This is achieved by providing independent authentication and privacy stings between $v^{\prime }$ and S. First, $\rho_{I}$ based assessments provide $\left[G,t,\hat{S}\right]$ features in $A_{prim}$ for security, the “t”. Pursued by this process, $P\left(t\right)$ in $S_{prim}$ retains the session endurance until ∆R is received. Therefore, there is a change in the different verification phases for (t) and G−G, as in Equation (5). The validation is performed for $A_{prim}$ in t and $S_{prim}$ in $\left(t+1\right)$ for Pdt such that $P_{I}=0$ or $P_{S}=0$ is identified. If this is identified, a new I will be allocated for ∆R, and the service will be retained. Contrarily, if ∆R is not achievable, then the privacy of V is retained, preventing further R failures. Thus the ${⨅}_{P-D}$ or ${⨅}_{P-G}$ is decided to communicate “t” further. This improves the session’s endurance, reducing the adversary’s impact. Similarly, the state analysis in Equation (8) determines the requirement or end of a “t”. The transaction requiring V is disconnected from the session, so the communicating “t” is retained. This prevents false transmissions and pauses “t”, maximizing the endurance. A comparative analysis for service endurance is presented in Figure 11.

### 4.6. Communication Cost

**Figure 12:**
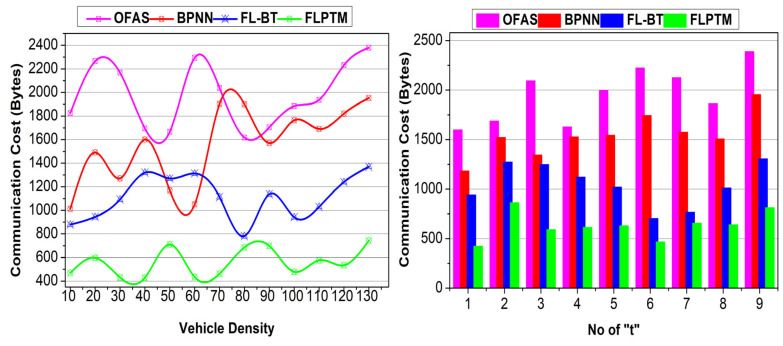
Communication cost comparisons.

Including adversaries in a “t” requires altering the session and a new I for communication. This requirement is reduced in the proposed scheme by performing two different assessments. First, the validation is preceded based on privacy maximizing $\frac{∆R}{R}$. The $\hat{S}$ is defined as high for service access, so the dissemination is masked above the required α. This is verified until $\rho_{I}-\rho_{S}=1$ is satisfied. Contrarily $A_{prim}$ ensures a reliable communication with $\rho_{I}=0$. Therefore, an additional requirement for the “t” is not mandatory, pursuing the ${⨅}_{G-G}$. This ensures no additional control data between Vs and Is. The second validation is the state identification defined through Equation (7). The $\frac{∆R}{R}$ maximization is required for $\rho_{I}=0$ or 1 without increasing the adversary impact. In Equation (8), transaction validation is performed to balance multiple ∆R constraints and reduce the false rate. The v is revoked from the communication provided τ≠0 and t<Pdt in the $\rho_{S}=0$ condition. This requires some communication message to be shared between the vs or Is to establish communication. In the overall process, the revocation confines additional control messages, reducing communication costs (refer to Figure 12).

The summary of these comparative findings for the FLPTM system has proven it to be the most effective approach in decreasing access time, lowering adversary impact, enhancing response time, boosting service durability, and cutting communication costs. It continuously beats all other methods across all parameters. Based on these findings, the FLPTM system is the go-to protocol for real-time traffic management and communication since it is the most efficient and secure alternative for smart transportation systems. Table 3 presents the comparative analysis results based on the above discussion.

The parameter ‘t’ is used in two different contexts: First, as a temporal measure, it indicates the elapsed time while the system is operating or the data are under analysis. The second use of ‘t’ is an iteration index showing the number of training or computational cycles carried out in the federated learning process. In such cases, the exact meaning of ‘t’ will be specified in the context. For example,‘t’ represents time in seconds for the performance analysis metrics but not the number of iterations during the federated aggregation steps.

## 5. Conclusions

An innovative ITS architecture, the proposed FLPTM system, analyses state coalition representation to provide efficient and secure authentication in a dynamic vehicular network. The CPPS framework combines a classifier-based learning technique with state modelling and access permissions to protect user data against invasions and man-in-the-middle assaults. The model includes FL to improve data security across transactions by facilitating updates that preserve privacy, allowing collaborative learning without revealing raw data, and so on. It eliminates problems like communication cost, adversary impact, and access time, cutting costs by 23.29%, 16.1%, and 18.95%, respectively. To further prove its resistance against privacy leaks, CPPS minimizes reliance on fixed data points and employs partial privacy protections. As a key component of intelligent transportation systems, it may greatly improve service durability by facilitating high-volume, continuous data transfers. Improved classifier adaptability to complex attack vectors, advanced machine learning integration, CPPS expansion to cover more vehicle data types, communication cost optimization using lightweight cryptographic methods or blockchain, and real-world traffic evaluation to find performance and scalability issues could be future work.

## Figures and Tables

**Table 1:** G for different “t”.

“t”	$\Gamma_{P-D}$	$\Gamma_{P-G}$	Service Endurance (%)	G
1	0	43	98.3	1
2	5	36	96.45	0.96
3	3	40	91.58	0.69
4	6	38	93.21	0.841
5	8	25	90.56	0.73
6	9	14	89.36	0.58
7	10	15	87.45	0.43
8	11	8	84.91	0

**Table 2:** Service endurance and communication cost.

Vehicle Density	Access Grant	Service Failure (%)	Communication Cost (Bytes)	Service Endurance (%)
20	1	0	410	98.3
40	0.95	3.36	639	97.02
60	0.81	5.69	931	95.4
80	0.73	9.48	1523	91.26
100	0.62	12.54	1958	89.58
120	0.43	15.3	2394	84.91

**Table 3:** Comparative analysis results for vehicle density and no. of “t”.

Metrics	OFAS	BPNN	FL–PT	FLPTM	Findings
Vehicle Density					
Access Time (ms)	1272.62	915.39	549.38	315.035	21.82%
Adversary Impact	0.863	0.657	0.336	0.1265	16.41%
Response Time (ms)	1612.71	1246.06	813.6	480.508	20.25%
Service Endurance (%)	86.74	88.29	90.11	93.597	15.65%
Communication Cost (Bytes)	2379	1953	1369	745	20.3%
No. of “**t**”					
Access Time (ms)	1284.05	1023.71	798.5	312.107	23.29%
Adversary Impact	0.798	0.625	0.384	0.12	16.1%
Response Time (ms)	1640.67	1281.24	848.86	593.816	17.59%
Service Endurance (%)	84.06	87.47	92.15	94.301	19.2%
Communication Cost (Bytes)	2389	1955	1307	813	18.95%

## Data Availability

The authors will make the data available upon request.

## Nomenclature

Parameter	Description
V	Vehicles
R	Requests
t	Time Interval
∆R	Response
s	Available service providers
S	Infrastructure
$\rho_{I}$ and $\rho_{S}$	Availability of infrastructure and service providers
G	Grant process
P	Permission
$\hat{S}$	Service level
$\alpha_{G}$	Grant state
$\alpha_{D}$	Deny state
$\alpha_{p}$	Pending state
$\prod_{P-D}\;and\;\prod_{P-G}$	Transaction for the appropriate states
$A_{prim}$and $S_{prim}$	Vehicle’s and service providers’ primitives for privacy
k	Non-replicated key
α	Random generator
p(t)	Probability
pdt	Probability that a valid service request
G⋅p dt	Instantaneous probability density of granting access
τ	Failure probability
ρ	The fraction of system resources available to serve requests

## References

[1] Hashem I.A., Siddiqa A., Alaba F.A., Bilal M., Alhashmi S.M. (2024). Distributed intelligence for IoT-based smart cities: A survey. Neural Comput. Appl..

[2] Selvaraj R., Kuthadi V.M., Duraisamy A., Selvaraj B., Pethuraj M.S. (2023). Learning Optimizer-Based Visual Analytics Method to Detect Targets in Autonomous Unmanned Aerial Vehicles. IEEE Intell. Transp. Syst. Mag..

[3] Alladi T., Kohli V., Chamola V., Yu F.R. (2023). A deep learning based misbehavior classification scheme for intrusion detection in cooperative intelligent transportation systems. Digit. Commun. Netw..

[4] Xu H., Yuan J., Berres A., Shao Y., Wang C., Li W., Wang H. (2023). A mobile edge computing framework for traffic optimization at urban intersections through cyber-physical integration. IEEE Trans. Intell. Veh..

[5] Vallent T.F., Hanyurwimfura D., Mikeka C. (2021). Efficient Certificate-Less Aggregate Signature Scheme with Conditional Privacy-Preservation for Vehicular Ad Hoc Networks Enhanced Smart Grid System. Sensors.

[6] Alaya B., Sellami L. (2023). Toward the Design of an Efficient and Secure System Based on the Software-Defined Network Paradigm for Vehicular Networks. IEEE Access.

[7] AlMarshoud M., Sabir Kiraz M., HAl-Bayatti A. (2024). Security, privacy, and decentralized trust management in VANETs: A review of current research and future directions. ACM Comput. Surv..

[8] Jiang S., Cao J., Wu H., Chen K., Liu X. (2023). Privacy-preserving and efficient data sharing for blockchain-based intelligent transportation systems. Inf. Sci..

[9] Mikavica B., Kostić-Ljubisavljević A. (2021). Blockchain-based solutions for security, privacy, and trust management in vehicular networks: A survey. J. Supercomput..

[10] Hbaieb A., Ayed S., Chaari L. (2022). A survey of trust management in the Internet of Vehicles. Comput. Netw..

[11] Yu Y., Xue X., Ma J., Zhang E.Z., Guan Y., Lu R. (2023). Efficient Privacy-Preserving Task Allocation with Secret Sharing for Vehicular Crowdsensing. IEEE Internet Things J..

[12] Yang M., Guo T., Zhu T., Tjuawinata I., Zhao J., Lam K.-Y. (2023). Local differential privacy and its applications: A comprehensive survey. Comput. Stand. Interfaces.

[13] Yao A., Li G., Li X., Jiang F., Xu J., Liu X. (2023). Differential privacy in edge computing-based smart city Applications: Security issues, solutions and future directions. Array.

[14] Liu Y., Zhang Y., Su S., Zhang L., Du X., Guizani M., Tian Z. (2023). BlockSC: A Blockchain Empowered Spatial Crowdsourcing Service in Metaverse While Preserving User Location Privacy. IEEE J. Sel. Areas Commun..

[15] Dzemydienė D., Burinskienė A., Čižiūnienė K., Miliauskas A. (2023). Development of E-Service Provision System Architecture Based on IoT and WSNs for Monitoring and Management of Freight Intermodal Transportation. Sensors.

[16] Zhang S., Li J., Shi L., Ding M., Nguyen D.C., Tan W., Weng J., Han Z. (2023). Federated Learning in Intelligent Transportation Systems: Recent Applications and Open Problems. IEEE Trans. Intell. Transp. Syst..

[17] Kaleem S., Sohail A., Tariq M.U., Asim M. (2023). An improved big data analytics architecture using federated learning for IoT-enabled urban intelligent transportation systems. Sustainability.

[18] Oladimeji D., Gupta K., Kose N.A., Gundogan K., Ge L., Liang F. (2023). Smart Transportation: An Overview of Technologies and Applications. Sensors.

[19] Shim K.A. (2023). Security Analysis of Conditional Privacy-Preserving Authentication Schemes for VANETs. IEEE Access.

[20] Kumari M., Ulmas Z., Suseendra R., Ramesh N., Venkata J., Baker El-Ebiary Y.A. (2024). Utilizing Federated Learning for Enhanced Real-Time Traffic Prediction in Smart Urban Environments. Int. J. Adv. Comput. Sci. Appl..

[21] Munawar A., Piantanakulchai M. (2024). A collaborative privacy-preserving approach for passenger demand forecasting of autonomous taxis empowered by federated learning in smart cities. Sci. Rep..

[22] Huang X., Huang T., Gu S., Zhao S., Zhang G. (2024). Responsible Federated Learning in Smart Transportation: Outlooks and Challenges. arXiv.

[23] Moulahi T., Jabbar R., Alabdulatif A., Abbas S., El Khediri S., Zidi S., Rizwan M. (2023). Privacy-preserving federated learning cyber-threat detection for intelligent transport systems with blockchain-based security. Expert Syst..

[24] Hu S., Ye Y., Hu Q., Liu X., Cao S., Yang H.H., Wu C. (2023). A Federated Learning-Based Framework for Ride-Sourcing Traffic Demand Prediction. IEEE Trans. Veh. Technol..

[25] Shen Y., Shen S., Li Q., Zhou H., Wu Z., Qu Y. (2023). Evolutionary privacy-preserving learning strategies for edge-based IoT data sharing schemes. Digit. Commun. Netw..

[26] Alshudukhi J.S., Al-Mekhlafi Z.G., Mohammed B.A. (2021). A Lightweight Authentication with Privacy-Preserving Scheme for Vehicular Ad Hoc Networks Based on Elliptic Curve Cryptography. IEEE Access.

[27] Chougule A., Chamola V., Hassija V., Gupta P., Yu F.R. (2023). A Novel Framework for Traffic Congestion Management at Intersections Using Federated Learning and Vertical Partitioning. IEEE Trans. Consum. Electron..

[28] Huang H., Hu Z., Wang Y., Lu Z., Wen X., Fu B. (2023). Train a central traffic prediction model using local data: A spatio-temporal network based on federated learning. Eng. Appl. Artif. Intell..

[29] Soleimany A., Farhang Y., Babazadeh Sangar A. (2023). Hierarchical federated learning model for traffic light management in future smart. Int. J. Nonlinear Anal. Appl..

